# Selection criteria for assembling a pediatric cancer predisposition syndrome gene panel

**DOI:** 10.1007/s10689-021-00254-0

**Published:** 2021-06-01

**Authors:** Anna Byrjalsen, Illja J. Diets, Jette Bakhuizen, Thomas van Overeem Hansen, Kjeld Schmiegelow, Anne-Marie Gerdes, Ulrik Stoltze, Roland P. Kuiper, Johannes H. M. Merks, Karin Wadt, Marjolijn Jongmans

**Affiliations:** 1 Department of Clinical Genetics, Rigshospitalet, Blegdamsvej 9, 2100 Copenhagen East, Denmark; 2 Department of Human Genetics, Radboudumc, Geert Grooteplein Zuid 10, 6525 GA Nijmegen, The Netherlands; 3 Princess Máxima Center for Pediatric Oncology, Heidelberglaan 25, 3584 CS Utrecht, The Netherlands; 4 Department of Genetics, University Medical Center Utrecht, 3508 AB Utrecht, The Netherlands; 5 Department of Paediatrics and Adolescent Medicine, Rigshospitalet, Blegdamsvej 9, 2100 Copenhagen East, Denmark

**Keywords:** Childhood cancer predisposition syndrome, Gene panel, Gene selection, Pediatric cancer, Gene selection, Genetic predisposition

## Abstract

Increasing use of genomic sequencing enables standardized screening of all childhood cancer predisposition syndromes (CPS) in children with cancer. Gene panels currently used often include adult-onset CPS genes and genes without substantial evidence linking them to cancer predisposition. We have developed criteria to select genes relevant for childhood-onset CPS and assembled a gene panel for use in children with cancer. We applied our criteria to 381 candidate genes, which were selected through two in-house panels (n = 338), a literature search (n = 39), and by assessing two Genomics England’s PanelApp panels (n = 4). We developed evaluation criteria that determined a gene’s eligibility for inclusion on a childhood-onset CPS gene panel. These criteria assessed (1) relevance in childhood cancer by a minimum of five childhood cancer patients reported carrying a pathogenic variant in the gene and (2) evidence supporting a causal relation between variants in this gene and cancer development. 138 genes fulfilled the criteria. In this study we have developed criteria to compile a childhood cancer predisposition gene panel which might ultimately be used in a clinical setting, regardless of the specific type of childhood cancer. This panel will be evaluated in a prospective study. The panel is available on (pediatric-cancer-predisposition-genepanel.nl) and will be regularly updated.

**Supplementary Information:**

The online version contains supplementary material available at 10.1007/s10689-021-00254-0.

## Introduction

Cancer affects approximately 15,000 children and 20,000 young adults in Europe each year [1]. In a significant percentage of cases, the cancer is caused by a pathogenic germline variant in a cancer predisposition gene. In recent studies, this percentage is estimated to be 7−10% [2–4]. In many of these patients the cancer predisposition syndrome (CPS) is recognizable by additional features or a positive family history. However, genetic predisposition might also play a role in children without recognizable features of genetic predisposition. For example, in patients with pathogenic de novo variants in the *TP53* gene, causing Li-Fraumeni syndrome (LFS), a family history of cancers suggesting the LFS diagnosis will be absent [5].

Many pediatric cancer centers have introduced whole exome or genome sequencing (WES, WGS) in the diagnostic work-up of pediatric cancer aiming to further personalize therapy. In clinical practice, tumor DNA is sequenced, while DNA derived from blood, or another tissue in the case of hematological cancer, is used as a germline reference. This, termed tumor-normal sequencing, enables the identification of acquired, i.e. somatic, cancer-driving variants [6, 7]. The availability of WES or WGS data from non-malignant tissues provides the opportunity to interrogate this data when there is a clinical suspicion of a CPS for which a molecular confirmation is required. In addition, it raises opportunities to perform germline genetic analysis in all patients, regardless of features suggestive of a CPS.

Recognition of cancer susceptibility in children with cancer can be of high clinical significance. It may lead to modifications in treatment protocols, inform the prognosis, and allow for tumor surveillance for early detection of second primary malignancies. In addition, it will enable genetic counseling and testing of family members with the option of offering cancer surveillance to at-risk individuals. Also, detection of a CPS might trigger awareness for other clinical manifestations related to the syndrome, for instance a congenital heart defect. Therefore, both physicians and parents are often in favor of seizing the opportunity to perform broad screening for cancer predisposing variants. However, apart from resulting in health benefits, germline genetic testing in children with cancer sometimes results in feelings of distress and insecurity and detection of a CPS can have social implications later in life [8–10]. Furthermore, analysis of large numbers of genes involves a probability of identifying variants of unknown significance (VUSs) and/or secondary findings [11, 12]. The latter refers to detection of a pathogenic variant in a gene associated with a condition unrelated to the cancer observed in the child [13]. To minimize the probability of such findings, filters can be applied to restrict the analysis to genes with a known association to the genetic condition(s). Such a collection of relevant genes is typically called a gene panel, and these have been applied for multiple groups of conditions, including CPSs [14, 15].

Literature describing the application of gene panels in genetic diagnostics rarely elucidates the criteria used for the composition of the gene panel. In the field of childhood cancer predisposition, the gene panels have so far mainly been applied in research settings. These research panels are usually large, highly variable, and often contain numerous genes that only play a role in susceptibility to cancer in adulthood. Testing children for adult-onset cancer syndromes, however, is largely considered undesirable unless preventive actions can be initiated before adulthood [16]. In addition, these panels often contain genes for which the evidence linking the gene to cancer is too weak for application in a diagnostic setting [2, 7, 17–19]. This highlights the need for specific gene panels within pediatric oncology, which includes only the genes with an evidence-based causal relation with the phenotype.

We aimed to develop criteria to select genes eligible for inclusion in a childhood CPS gene panel. We applied these criteria to genes already present in panels that are currently used for CPS testing and we searched the literature for novel pediatric cancer predisposition genes. Finally, we discuss in which settings this panel can be applied and when to opt for targeted testing or smaller gene panels.

## Materials and methods

### Source of cancer predisposition panel

We selected candidate genes from two internal sources: the hereditary cancer gene panel from the Radboudumc (version DG2.17), containing 232 genes known to cause cancer in adults and children, and the gene list from the STAGING study (national research study performing WGS of childhood cancer patients in Denmark) (v2.0), containing 314 genes related to or potentially related to childhood cancer predisposition. These genes were originally chosen based on published gene lists [2, 17], from linkage to pediatric cancer in literature, conference abstracts and presentations attended by the researchers. Collectively, the Radboudumc and STAGING panels comprised 338 unique genes, 201 genes overlapped between the two panels. Genes are named according to the HUGO Gene Nomenclature Committee (HGNC) guidelines.

In addition, we performed a literature search in PubMed to identify CPS-genes not included in the two panels. This search combined search terms addressing the population in question (children), the diagnosis and inherited predisposition. The search terms used are listed in Supplementary file 1. Through this search, we identified an additional 39 genes which had been linked to childhood cancer predisposition in the literature.

Finally, we used the opensource PanelApp developed as part of the Genomics England project providing panels for use both in routine diagnostics and in research. This resource was specifically chosen because the PanelApp panels are meant for use in a diagnostic setting, while most other published panels are intended for use in a research setting. The PanelApp is an effort trying to include only genes with a certain amount of evidence supporting pathogenicity, as opposed to research panels which are often very broad and non-restrictive in the choice of genes included. We identified two gene panels with relevance to our study called “Tumour predisposition – childhood onset (Version 2.1)” and “Childhood solid tumours cancer susceptibility (Version 1.6)” [20], yielding 114 and 83 genes respectively. Combining these panels yielded 116 unique genes.

## Results

**Fig. 1:**
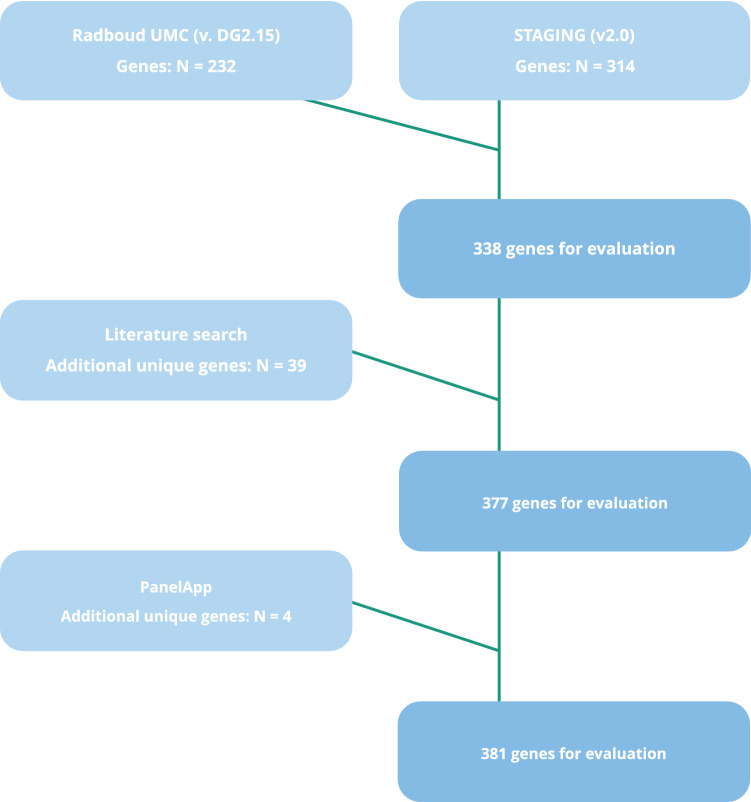
Sources for identification of candidate genes predisposing to childhood-onset CPS

Combining the genes identified through the Radboudumc- and the STAGING panel with the genes identified through the literature search yielded 377 unique genes. In addition, 4 candidate genes were identified through the PanelApp yielding in total 381 unique candidate genes (Fig. 1, Supplementary file 2).

### Defining criteria for inclusion

The criteria were developed by a binational expert panel, which included clinical geneticists, molecular biologists, and pediatric oncologists. In order to develop the criteria for selecting genes eligible for the Pediatric CPS gene panel, we divided the genes into two categories: Category 1 consisted of *individual genes*: genes causing a CPS that is not associated with variants in other genes, and Category 2 consisted of *group genes*: genes that can be grouped phenotypically together, as they give rise to the same CPS. Genes were evaluated solely on their gene-cancer association, and not their association with other conditions. Only pathogenic and likely pathogenic variants (class 5 and 4) were deemed relevant and included in our definitions. They will be collectively referred to as ‘pathogenic variants’ [21].

Category 1: We defined that for each gene a pathogenic variant in a minimum of five children (aged 0–18 at diagnosis) with a cancer diagnosis in at least two independent families should be reported in the literature (criteria A in Table 1). Also included among these genes predisposing to myelodysplastic syndrome (MDS), which is an established precursor of hematologic malignancies*.* This minimum number of cases was independent of the prevalence in adult-onset cancer patients and was applied to ensure that the gene was indeed predisposing to childhood-onset cancer. Previously published guidelines require that only 3 patients with a given phenotype carry a pathogenic variant in a gene [22]. We have chosen to apply a threshold of 5 described cases because we judge a proven relevance in childhood extremely important for eligibility to a gene panel that is used for diagnostic purposes (criteria B in Table 1). Furthermore, we anticipate that newly discovered genes that are currently just below this threshold will meet the criteria in the (near) future and will be added in an updated version. Furthermore, there should be additional evidence supporting the relation between pathogenic variants in the gene of interest and cancer development (criteria B in Table 1). This evidence can consist of segregation analysis in a family (e.g. for expected high-penetrance variants present in three or more affected relatives), tumor analysis demonstrating a second hit in the tumor, consistent somatic mutational signatures, animal studies supporting pathogenicity, or studies which support pathogenicity in organoid or other cellular models. The additional evidence was included to ensure that syndromes otherwise common in the population would not be included just because cancers would occur by chance in children with these syndromes (e.g. cystic fibrosis or phenylketonuria).

**Table 1:** Criteria for inclusion of a gene in the Pediatric CPS gene panel

1. For *individual genes*	
A. Reports of childhood cancer^a^ in the literature	Childhood cancer is reported in a minimum of 5 different children (< 18 years of age) with a pathogenic variant in the specific gene, from at least 2 different families
AND	
B. Additional evidence in the literature	A minimum of 1 study supporting a causal relation with cancer development is published (i.e. studies containing somatic second hits in the tumor, segregation in families (e.g. for expected high-penetrance variants present in three or more affected relatives), consistent somatic mutational signatures, animal studies supporting pathogenicity, or cellular/organoid models supporting pathogenicity)
2. For syndromes that have been associated with pathogenic variants in ≥ 2 different genes (*group genes*)^b^	
A. Reports of childhood cancer in literature	Two of the genes in the group fulfill inclusion as individual genes (1A AND 1B); OR More than 20 cases with a childhood cancer have been described in total with this syndrome and at least one of the genes fulfills 1B

^a^Including myelodysplastic syndrome

^b^All genes associated with the syndrome in the literature will be included if they share the same biologic pathway or mechanism. These syndromes are constitutional mismatch repair deficiency syndrome, Fanconi anemia, Diamond-Blackfan anemia, dyskeratosis congenita, hereditary paraganglioma-pheochromocytoma, mosaic variegated aneuploidy syndrome Noonan syndrome and Xeroderma pigmentosum

Category 2: For this category we defined several groups of disorders that can be caused by more than two different genes. These genes should have similar functions in the same biological pathway for it to be considered as a group. We included all genes in the group that cause the syndrome in the gene panel, as long as at least two of the genes fulfill the criteria for single genes or in which more than 20 cases have been reported in total (e.g. Diamond-Blackfan anemia, Table 1). The groups we defined included genes causing the conditions constitutional mismatch repair deficiency (CMMRD) and Lynch syndrome, Diamond-Blackfan anemia, dyskeratosis congenita, Fanconi anemia, Gorlin syndrome, hereditary paraganglioma-pheochromocytoma, mosaic variegated aneuploidy syndrome, Noonan/Noonan like syndrome, and xeroderma pigmentosum.

### Formation of the Pediatric CPS gene panel

We applied the criteria to all 381 genes and found that 64 genes were included through the single gene criteria (Category 1) and 74 genes fulfilled the group criteria (Category 2). In total, 138 genes were included in our panel, and 243 genes did not fulfill the criteria at this time (*ELP1* is a gene which fulfilled the criteria just prior to submission). The genes included in the panel are listed in Table 2. All genes we assessed can be found in Supplementary file 2 including references and number of patients identified.

**Table 2:** Genes included in the Pediatric CPS gene panel

*Genes included through the single gene criteria*^a^						
*ABCB11*	*CDH1*	*ETV6*	*ITK*	*PTEN*	*SETBP1*	*TSC2*
*AIP*	*CDKN1C*	*EZH2*	*LIG4*	*RB1*	*SH2D1A*	*USB1*
*ALK*	*CDKN2A*	*FAS*	*MEN1*	*RECQL4*	*SMARCA4*	*WAS*
*APC*	*CEBPA*	*FBXW7*	*NBN*	*REST*	*SMARCB1*	*WT1*
*ATM*	*CREBBP*	*GATA2*	*NF1*	*RET*	*SMARCE1*	
*BAP1*	*CTLA4*	*GPC3*	*NF2*	*RMRP*	*STK11*	
*BLM*	*CTR9*	*GPR161*	*NSD1*	*RUNX1*	*TP53*	
*CD27*	*DICER1*	*HAVCR2*	*PAX5*	*SAMD9*	*TRIM28*	
*CD70*	*DIS3L2*	*HRAS*	*PHOX2B*	*SAMD9L*	*TRIM37*	
*CDC73*	*ELP1*	*IKZF1*	*PIK3CA*	*SBDS*	*TSC1*^b^	
*Genes included through the group criteria*						
Constitutional mismatch repair deficiency/Lynch syndrome		***MLH1****, ****MSH2***^c^*, ****MSH6****, ****PMS2***				
Diamond-Blackfan anemia		*RPL5, RPL11, RPL35A, RPS10, RPS17, RPS19, RPS24, RPS26, RPS7*				
Dyskeratosis congenita		*ACD, CTC1, DKC1, NHP2, NOP10, PARN, RTEL1, TERC, TERT, TINF2, WRAP53*				
Fanconi anemia		***BRCA2, BRIP1, ERCC4***^d^*, ****FANCA, FANCB, FANCC, FANCD2, FANCE, FANCF, FANCG, FANCI, FANCL, PALB2***				
Gorlin syndrome		***PTCH1, PTCH2, SUFU***				
Hereditary paraganglioma–pheochromocytoma, (genes following the same pathway^e^)		***FH, EPAS1 (HIF2A), MDH2, EGLN1 (PHD2), EGLN2 (PHD1), SDHA****, ****SDHB****, ****SDHC****, ****SDHD, SDHAF2, VHL***				
Mosaic variegated aneuploidy syndrome		***BUB1B****, ****TRIP13, CEP57***				
Noonan (like) syndrome		*A2ML1, BRAF, ****CBL, KRAS, LZTR1, MAP2K1, MAP2K2, NRAS, PTPN11, RAF1, RIT1, SHOC2, SOS1***				
Xeroderma pigmentosum		*DDB2, ERCC2, ERCC3, ERCC4*^*4*^*, ERCC5, POLH, XPA, XPC*				

Genes in bold also fulfill Category 1 for individual genes

Variants in the mismatch repair genes, *PALB2*, *BRCA2 and ATM* will be reported both in a heterozygous and bi-allelic state

For Diamond-Blackfan anemia, dyskeratosis congenita and xeroderma pigmentosum publications report cases without differentiating which gene is affected

^a^For full gene list see supplementary material

^b^Included despite not fulfilling the single gene criteria, as it causes the same phenotype as *TSC2*, which fulfils the criteria

^c^Including 3′ end deletions of *EPCAM* resulting in allele-specific epigenetic silencing of *MSH2*^23^

^d^Causing both Fanconi anemia and xeroderma pigmentosum

^e^Other genes also cause hereditary paraganglioma-pheochromocytoma but follow a different pathway, and did not fulfill the group criteria

Finally, we compared the Pediatric CPS gene panel to the two pediatric cancer gene panels available in the Genomics Englands’ PanelApp (“Tumour predisposition—childhood onset (Version 2.1)” and “Childhood solid tumours cancer susceptibility panel (Version 1.6)”) [20]. We combined the two panels, since they largely overlap, resulting in 116 unique genes (Supplementary file 2). The PanelApp uses a traffic light system to illustrate the evidence supporting the presence of a gene in the panel. Genes are marked either red, orange or green. Green indicates that pathogenic variants in this gene were found in three or more unrelated cases/families with the given disorder or from 2 to 3 unrelated cases/families, and the availability of supporting functional data for the causal relation. Genes that almost meet these criteria are rated as orange, whereas red genes have a low level of evidence supporting association [19].

**Fig. 2:**
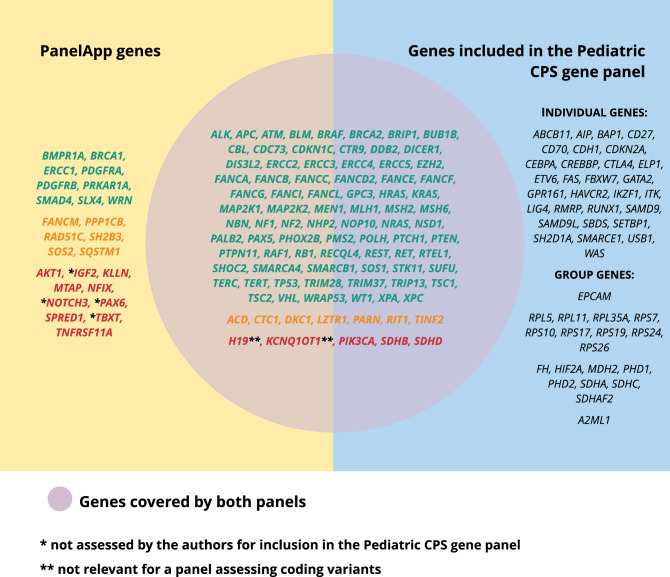
Evaluation of the genes included in the two Genomics England panels (“Tumour predisposition – childhood onset (Version 2.1)” and “Childhood solid tumours cancer susceptibility panel (Version 1.6)”) according to the Pediatric CPS gene panel. Green genes: pathogenic variants found in three or more unrelated cases/families with the given disorder or from 2 to 3 unrelated cases/families and the availability of supporting functional data. Orange genes: genes which almost meet the criteria listed above. Red genes: low level of evidence supporting association

The two panels included 88 green genes, 13 orange genes and 15 red genes. Only four genes, all marked red (*IGF2*, *NOTCH3*, *PAX6*, *TBXT*), were not in our list of candidate genes and none of these genes fulfilled our criteria for inclusion in the Pediatric CPS gene panel. In addition, nine genes marked as green also did not fulfill our criteria and therefore were not included (*BMPR1A*, *BRCA1*, *ERCC1*, *PDGFRA*, *PDGFRB*, *PRKAR1A*, *SMAD4, SLX4* and *WRN*). Finally, the Pediatric CPS gene panel encompasses 48 genes not mentioned in the panels from Genomics Englands’ PanelApp (Fig. 2).

## Discussion

Here, we present criteria to select genes eligible for the Pediatric CPS gene panel and the genes that currently fulfill these criteria. The need for these criteria is urgent since broad screening for germline cancer predisposing variants in children with cancer is gradually shifting from a research setting to routine diagnostics. The Pediatric CPS gene panel will aid the identification of genetic variants within genes with a proven causal link to childhood cancer which is a prerequisite in a diagnostic setting.

Our criteria ensure selection of genes that are truly involved in pediatric cancer, by using a minimal threshold of five reported cases of cancer in childhood. This is higher than what has been used in other studies [22]. The genes involved in Kabuki syndrome, *KMTD2* and *KDM6A*, which are affected by somatic inactivating variants in sporadic cancers, are an example of genes that were excluded based on this threshold. Some adult CPS genes are included in the gene panel described here. These are genes that can cause a childhood CPS if both alleles are affected, whereas cancer in adulthood has been linked to heterozygous variants in these genes. An example is the *ATM* gene that is involved in ataxia teleangiectasia in children and susceptibility to breast cancer in women.

For several adult CPS genes, sequencing studies revealed an enrichment of heterozygous pathogenic germline variants in children with cancer [2, 23]. Such potential associations need evaluation and confirmation before genes are routinely sequenced in children. This requires for sequencing data from a large number of childhood cancer patients in multicenter studies and subsequent studies to validate findings and support causality. An example of a study supporting such causality showed that heterozygous pathogenic germline variants in *PALB2* and *BRCA2* in children with medulloblastoma had somatic mutational signatures typical of *PALB2*/*BRCA2*-related homologous recombination repair deficiency in the corresponding tumors [24].

Application of these criteria reduced the number of genes in a general cancer predisposition panel (Radboudumc DG2.17) from 232 to 126 genes that are specifically relevant to a childhood cancer population. By investigating fewer genes, the number of variants requiring further investigation as well as the number of incidental findings will decrease. A consequence of our stringent criteria may be that CPS with low penetrance in childhood might be missed. Nevertheless. If these genes have a true correlation with cancer, this will result in additional cases and the genes will be added to the panel at a later moment.

In comparing the Pediatric CPS gene panel with the PanelApp panels “Tumour predisposition—childhood onset (Version 1.36)” and “Childhood solid tumours cancer susceptibility (Version 1.6)”, which are meant for application in a diagnostic setting and updated regularly, we observed quite some differences. Some of these are explained by inclusion of genes in our panel by the group criteria, whereas PanelApp states explicitly that only genes with strong evidence of association with cancer are included as’Green’ on this panel for genetically heterogeneous conditions such as Fanconi anemia. Furthermore, the PanelApp panel ‘Childhood solid tumours cancer susceptibility’ has a disclaimer that the panel includes genes predisposing to childhood tumors not otherwise covered by a more specific panel, whereas our aim was to include all CPS genes. Additionally, PanelApp advises that their panel should be applied to cancer patients < 25 years of age while our target group is children < 18 years of age. Taking these differences into account, we still noted that several genes were missing in the PanelApp panels which most likely would fulfill their criteria, for instance the leukemia predisposing genes *ETV6* and *RUNX1* (not mentioned) and the hereditary paraganglioma gene *SDHB* (marked red).

Now that we have developed the Pediatric CPS gene panel, the next question is when to apply it in clinical care. The answer to this question is not necessarily the same for each center and depends on local circumstances. These include the availability of laboratory facilities and techniques, funding by health insurances and local opinions regarding application of broad genetic testing in children which can be influenced by culture and religion. In general, a targeted analysis of a gene of interest in a child with features very specific for one particular genetic syndrome is sufficient to find molecular confirmation for the evident syndrome. Furthermore, for some malignancies smaller panels to test for germline causative variants are available. In particular this is the case for some ‘adult types’ of cancer that rarely occur in children, like gene panels addressing all melanoma predisposition genes or all colorectal cancer predisposition genes. One needs to be aware, however, that *TP53* is often missing in these adult CPS panels, whereas it is an important causative factor in adult types of cancer in children (e.g. melanoma, stomach carcinoma, and lung cancer) [25]. Finally, as broader NGS panels are now increasingly price-competitive, another drawback of using targeted/smaller panels is that these are not cost effective to use, particularly if the patient is not diagnosed in the first round of testing.

Our expectation is that application of a gene panel containing all pediatric CPS genes will particularly improve identification of syndromes in patients that lack additional characteristics suggestive of a constitutional variant or otherwise have a very mild presentation of a syndrome. To study this hypothesis, we will apply the Pediatric CPS gene panel prospectively to an unselected cohort of children with cancer in the Netherlands. We will compare the performance of the Pediatric CPS gene panel to the path of referral to a clinical geneticist of selected patients if a genetic syndrome is suspected. In this latter route, the pediatric oncologists will be supported in selecting patients for referral by using the McGill Interactive Pediatric OncoGenetic Guidelines (MIPOGG) criteria [26], which is an app-based tool that helps clinicians identify patients with a higher likelihood of harboring an underlying CPS.

If no causative variant is identified in the gene panel after analysis including a proper screen for copy number alterations, and suspicion for genetic predisposition is high, one can consider proceeding with an exome- or genome wide analysis. Previously, we showed that including parental DNA, which improves detection of de novo variants and variants that follow recessive inheritance, is very efficient with WES [23]. Of course, one needs to be aware that WES and WGS can miss syndromes caused by for instance aberrant methylation or mosaic variants. Optimal diagnostics therefore requires an integrated approach of clinical evaluation and genetic testing ensuring that clinical symptoms can direct further testing if initial results are negative.

Prior to applying a large CPS gene panel to children with cancer, the team providing the test needs to decide on the type of variants that will be reported—traditionally pathogenic variants (class 4 and 5). In our practice, heterozygous carrier status of pathogenic variants purely causing conditions inherited through an autosomal recessive inheritance pattern, will only be reported if the parents of the child are consanguineous. Variants of unknown significance (ACMG class 3) will not be reported, unless segregation analysis or other additional tests can potentially upgrade the variant to a class 4 or 5. We advise decisions regarding reporting of findings to be discussed at a tumor board prior to reporting.

Another prerequisite for applying a large CPS panel is of course proper genetic counseling aimed at preparing parents for these potential outcomes. This is doable with counselling provided by doctors or genetic counselors who have specialized within this field. We have recently shown that parents overall are keen to participate in genetic research of children with cancer [27], and that most agree to have findings in cancer genes reported back to them.

In this study we have developed criteria to compile the Pediatric CPS gene panel which might ultimately be used in a clinical setting, regardless of the specific type of childhood cancer. The Pediatric CPS gene panel will be evaluated in a prospective study, is available on pediatric-cancer-predisposition-genepanel.nl and will be regularly updated.

## Supplementary Information

Below is the link to the electronic supplementary material.

Supplementary file1 (DOCX 17 kb)

Supplementary file2 (XLSX 96 kb)
