# Supplementary material for: Selection criteria for assembling a pediatric cancer predisposition syndrome gene panel
Source: Fam Cancer. 2021 Jun 1;20(4):279–87. doi: 10.1007/s10689-021-00254-0 (PMC8484084; doi:10.1007/s10689-021-00254-0)
Supplement: Supplementary file 1 — Supplementary file1 (DOCX 17 kb) [file 10689_2021_254_MOESM1_ESM.docx]

**Supplementary File 1**

| **Search terms - PubMed** | | | | | |
| --- | --- | --- | --- | --- | --- |
| **Population** | | **Diagnosis** | | **Genetics** | |
| Mesh | Text word | Mesh | Text word | Mesh | Text word |
| **Infant** | Pediatric | **Neoplasms** |  | **Genome** | Genes |
| **Child** | Paediatric | Adrenocortical carcinoma | Adrenocortical carcinoma | **Genetics** | Genetics |
| **Adolescent** | Child | Astrocytoma | Astrocytoma | **Genotype** | Genetic predisposition to disease |
|  | Children | Central nervous system | Central nervous system | Genetic predisposition to disease | Genetic predisposition |
|  | Childhood |  | CNS tumor | **Genetic variation** | Genetic variation |
|  | Youth |  | Germ-cell tumor |  | Pathogenic mutation |
|  | Young | Optic nerve glioma | Optic nerve glioma |  | Novel genes |
|  | Juvenile | Glioma | Glioma |  | Oncogenes |
|  | Adolescent | Histiocytosis, Langerhans-Cell | LCH |  | Hereditary |
|  | Adolescence |  | Langerhans- cell histiocytosis |  | Inherited |
|  | Infant | Hepatoblastoma | Hepatoblastoma |  | Variant |
|  | Infancy |  | Kidney neoplasms |  | Pathogenic variant |
|  |  |  | Renal cancer |  | Germline |
|  |  | Leukemia | Leukemia |  | Germ-line |
|  |  | Lymphoma | Lymphoma |  | Mutation |
|  |  | Hodgkin Disease | Hodgkin lymphoma |  | Tumor suppressor gene |
|  |  |  | Hodgkin disease |  |  |
|  |  |  | Hodgkin |  |  |
|  |  |  | Non-Hodgkin lymphoma |  |  |
|  |  | Medulloblastoma | Medulloblastoma |  |  |
|  |  |  | Malignancy |  |  |
|  |  | Melanoma | Melanoma |  |  |
|  |  |  | Neoplasms |  |  |
|  |  |  | Cancer |  |  |
|  |  |  | Carcinoma |  |  |
|  |  |  | Malignant peripheral nerve sheath tumor |  |  |
|  |  | Neuroblastoma | Neuroblastoma |  |  |
|  |  | Pheochromocytoma | Pheochromocytoma |  |  |
|  |  | Retinoblastoma | Retinoblastoma |  |  |
|  |  | Rhabdoid tumor | Rhabdoid tumor |  |  |
|  |  |  | Rhabdoid tumor/teratoid tumor |  |  |
|  |  |  | ATRT |  |  |
|  |  | Rhabdomyosarcoma | Rhabdomyosarcoma |  |  |
|  |  | Sarcoma | Ewing sarcoma |  |  |
|  |  |  | Osteosarcoma |  |  |
|  |  |  | Tumor |  |  |
|  |  | Teratoid tumor | Teratoid tumor |  |  |
|  |  |  | Thyroid neoplasm |  |  |
|  |  |  | Thyroid cancer |  |  |
|  |  | Wilms tumor | Wilms tumor |  |  |

| **Search strategy** | | |
| --- | --- | --- |
| Database | Search string | Number of hits |
| **Pubmed**  Covering: 1966-Mar 2^nd^ 2020  Search date: Mar 2^nd^ 2020 | **#1** Population: Search (((Infant[MeSH Terms]) OR Child[MeSH Terms]) OR Adolescent[MeSH Terms]) OR ((Pediatric[Text Word] OR Paediatric[Text Word] OR Child[Text Word] OR Children[Text Word] OR Childhood[Text Word] OR Youth[Text Word] OR Young[Text Word] OR Juvenile[Text Word] OR Adolescent[Text Word] OR Adolescence[Text Word] OR Infant[Text Word] OR Infancy[Text Word])) | 4581017 |
|  | **#2** Diagnosis: Search (((((((((((((((((((((Neoplasm*[MeSH Terms]) OR Adrenocortical carcinoma[MeSH Terms]) OR Astrocytoma[MeSH Terms]) OR Central nervous system[MeSH Terms]) OR Optic nerve glioma[MeSH Terms]) OR Glioma[MeSH Terms]) OR Histiocytosis, Langerhans-Cell[MeSH Terms]) OR Hepatoblastoma[MeSH Terms]) OR Leukemia[MeSH Terms]) OR Lymphoma[MeSH Terms]) OR Hodgkin disease[MeSH Terms]) OR Medulloblastoma[MeSH Terms]) OR Melanoma[MeSH Terms]) OR Neuroblastoma[MeSH Terms]) OR Pheochromocytoma[MeSH Terms]) OR Retinoblastoma[MeSH Terms]) OR Rhabdoid tumor[MeSH Terms]) OR Rhabdomyosarcoma[MeSH Terms]) OR Sarcoma[MeSH Terms]) OR Teratoid tumor[MeSH Terms]) OR Wilms tumor[MeSH Terms]) OR (("Adrenocortical carcinoma"[Text Word] OR Astrocytoma[Text Word] OR "Central nervous system"[Text Word] OR "CNS tumor"[Text Word] OR "Germ-cell tumor"[Text Word] OR "Optic nerve glioma"[Text Word] OR Glioma[Text Word] OR LCH[Text Word] OR "Langerhans-cell histiocytosis"[Text Word] OR Hepatoblastoma[Text Word] OR "Kidney neoplasms"[Text Word] OR "Renal cancer"[Text Word] OR Leukemia[Text Word] OR Lymphoma[Text Word] OR "Hodgkin lymphoma"[Text Word] OR "Hodgkin disease"[Text Word] OR Hodgkin[Text Word] OR "Non-Hodgkin lymphoma"[Text Word] OR Medulloblastoma[Text Word] OR Malignancy[Text Word] OR Melanoma[Text Word] OR Neoplasm[Text Word] OR Cancer[Text Word] OR Carcinoma[Text Word] OR "Malignant peripheral nerve sheath tumor"[Text Word] OR Neuroblastoma[Text Word] OR Pheochromocytoma[Text Word] OR Retinoblastoma[Text Word] OR "Rhabdoid tumor"[Text Word] OR "Rhabdoid tumor/teratoid tumor"[Text Word] OR ATRT[Text Word] OR Rhabdomyosarcoma[Text Word] OR "Ewing sarcoma"[Text Word] OR Osteosarcoma[Text Word] OR Tumor[Text Word] OR "Teratoid tumor"[Text Word] OR "Thyroid neoplasm"[Text Word] OR "Thyroid cancer"[Text Word] OR "Wilms tumor"[Text Word])) | 5741357 |
|  | **#3** Genetics: Search (((((Genome[MeSH Terms]) OR Genetics[MeSH Terms]) OR Genotype[MeSH Terms]) OR genetic predisposition to disease[MeSH Terms]) OR Genetic variation[MeSH Terms]) OR ((Genes[Text Word] OR Genetic*[Text Word] OR "Genetic predisposition to disease"[Text Word] OR "Genetic predisposition"[Text Word] OR "Genetic variation"[Text Word] OR "Pathogenic mutation"[Text Word] OR "Novel gene"[Text Word] OR Oncogenes[Text Word] OR Hereditary[Text Word] OR Inherited[Text Word] OR Variant[Text Word] OR "Pathogenic variant"[Text Word] OR Germline[Text Word] OR "Germ-line"[Text Word] OR Mutation[Text Word] OR "Tumor suppressor gene"[Text Word])) | 4406485 |
|  | **#4** Combined search  **#1** AND **#2** AND **#3** | 132420 |
| Limiting to publications from Jan 1^st^ 2019–Mar 2^nd^ 2020 | **#4** Combined search  **#1** AND **#2** AND **#3** | 5370 |
